# Urea Amendment Decreases Microbial Diversity and Selects for Specific Nitrifying Strains in Eight Contrasting Agricultural Soils

**DOI:** 10.3389/fmicb.2018.00634

**Published:** 2018-04-04

**Authors:** Christopher Staley, Florence Breuillin-Sessoms, Ping Wang, Thomas Kaiser, Rodney T. Venterea, Michael J. Sadowsky

**Affiliations:** ^1^The BioTechnology Institute, University of Minnesota, St. Paul, MN, United States; ^2^Department of Soil, Water, and Climate, University of Minnesota, St. Paul, MN, United States; ^3^Soil and Water Management Research Unit, United States Department of Agriculture-Agricultural Research Service, St. Paul, MN, United States; ^4^Department of Plant and Microbial Biology, University of Minnesota, St. Paul, MN, United States

**Keywords:** agriculture, microbial community, nitrogen cycle, soil, urea

## Abstract

Application of nitrogen (N) fertilizers, predominantly as urea, is a major source of reactive N in the environment, with wide ranging effects including increased greenhouse gas accumulation in the atmosphere and aquatic eutrophication. The soil microbial community is the principal driver of soil N cycling; thus, improved understanding of microbial community responses to urea addition has widespread implications. We used next-generation amplicon sequencing of the 16S rRNA gene to characterize bacterial and archaeal communities in eight contrasting agricultural soil types amended with 0, 100, or 500 μg N g^-1^ of urea and incubated for 21 days. We hypothesized that urea amendment would have common, direct effects on the abundance and diversity of members of the microbial community associated with nitrification, across all soils, and would further affect the broader heterotrophic community resulting in decreased diversity and variation in abundances of specific taxa. Significant (*P* < 0.001) differences in bacterial community diversity and composition were observed by site, but amendment with only the greatest urea concentration significantly decreased Shannon indices. Expansion in the abundances of members of the families *Microbacteriaceae*, *Chitinophagaceae*, *Comamonadaceae*, *Xanthomonadaceae*, and *Nitrosomonadaceae* were also consistently observed among all soils (linear discriminant analysis score ≥ 3.0). Analysis of nitrifier genera revealed diverse, soil-specific distributions of oligotypes (strains), but few were correlated with nitrification gene abundances that were reported in a previous study. Our results suggest that the majority of the bacterial and archaeal community are likely unassociated with N cycling, but are significantly negatively impacted by urea application. Furthermore, these results reveal that amendment with high concentrations of urea may reduce nitrifier diversity, favoring specific strains, specifically those within the nitrifying genera *Nitrobacter, Nitrospira*, and *Nitrosospira*, that may play significant roles related to N cycling in soils receiving intensive urea inputs.

## Introduction

Worldwide demand for nitrogen (N) fertilizer is projected to increase at a rate of approximately 1.6 Tg (160,000 metric tons) of N per year, with much of the increase expected to occur in China (18%), India (17%) and Latin America (18%) (FAO, 2015). Production of N fertilizer via the Haber-Bosch process accounts for approximately 45% of global terrestrial N_2_ fixation annually (Canfield et al., 2010). Due to its relative ease of transport and use, urea (CO[NH_2_]_2_) is the most dominant chemical form of N fertilizer used in the United States Corn Belt and across the world. Recent reporting by the United Nations Food and Agriculture Organization^1^ indicated that approximately 80% of the total N fertilizer used worldwide in 2014 (113 Tg N) was in the form of urea (FAO, 2017). It is well known that application of N fertilizers is a major source of reactive N in the environment and contributes to several deleterious ecological outcomes. Negative effects include increased greenhouse gas composition of the atmosphere, stratospheric ozone depletion due to nitrous oxide (N_2_O) emissions, water quality impairment due to nitrate (NO_3_^-^) entering ground and surface waters, and various impacts of atmospheric N deposition to terrestrial ecosystems (Galloway et al., 2003). The N fertilizer inputs to soil also support the increased production of cereals and other crops, which are needed to support a growing world population. Thus, improved understanding of the response of the soil microbial community, which drives N cycling, to urea addition has widespread implications and applications.

Once applied to soil, urea first undergoes hydrolytic reactions that produce ammonia (NH_3_) which is then converted to nitrite (NO_2_^-^) and NO_3_^-^ via nitrification processes (Prosser, 1990). These latter substrates can be further transformed to other by-products, including N_2_O, by several processes including chemo-denitrification, nitrification, heterotrophic denitrification, and nitrifier-denitrification (Venterea et al., 2012). Denitrification is carried out under anaerobic conditions by a large variety of heterotrophic taxa, but only a few, such as *Pseudomonas* spp. and *Alcaligenes* spp., are numerically dominant in soils (Firestone and Davidson, 1989; Shoda, 2017), as are other members of the Alpha-, Beta-, and Gamma-proteobacteria (Jones et al., 2008). During nitrification, ammonia-oxidizing bacteria (AOB), such as those in the genera *Nitrosomonas* and *Nitrosospira*, as well as others, convert NH_3_ to NO_2_^-^, followed by oxidation of NO_2_^-^ to NO_3_^-^ by nitrite-oxidizing bacteria (NOB), predominantly within the genera *Nitrobacter* and *Nitrospira* in soils (Robertson and Groffman, 2007; Klotz et al., 2011; Ruser and Schulz, 2015; Heil et al., 2016). Ammonia-oxidizing archaea (AOA) have also been reported, although their relative abundance and overall contribution to the formation of NO_2_^-^ vary based on the environment studied (Leininger et al., 2006; Di et al., 2009; Zhang et al., 2012; Giguere et al., 2015; Ouyang et al., 2016). Furthermore, members of the genus *Nitrospira* have recently been reported to be capable of oxidizing both NH_3_ and NO_2_^-^, in effect performing complete oxidation of NH_3_ to NO_3_^-^ (Daims et al., 2015; van Kessel et al., 2015).

Addition of N fertilizers to soil, in various chemical forms, has been shown to reduce heterotrophic respiration due to a variety of factors that may include soil acidification and impacts on carbon cycling (Janssens et al., 2010; Ramirez et al., 2010). It has been further postulated that increasing soil N availability may favor nitrogen-limited species that utilize carbon sources more efficiently (Ågren et al., 2001). Moreover, according to the subsidy-stress hypothesis, moderate levels of N addition may increase microbial diversity while greater amendment concentrations may have a more negative toxic effect resulting in decreased diversity (Odum et al., 1979; Odum, 1985). Changes in microbial community function and biomass in response to N addition have been characterized (Compton et al., 2004; Frey et al., 2004), and discrete changes in community composition are hypothesized to be soil-specific (Geisseler and Scow, 2014). Recent studies have reported how different soil types can respond differently to urea addition in their accumulation of intermediate N substrates (i.e., NO_2_^-^ and NH_3_) which are known to be toxic or inhibitory to a range of organisms, including AOB and NOB (Venterea et al., 2015; Breuillin-Sessoms et al., 2017). However, there remains a paucity of evidence regarding how the amendment of soil with N fertilizers, or with urea specifically, influences the soil microbial community at large.

Advances in next-generation sequencing have enabled an unprecedented assessment of bacterial diversity in a variety of environments (Sogin et al., 2006; Staley and Sadowsky, 2016). However, characterization of entire bacterial and archaeal communities in soils has remained a challenge due to a high degree of variability across very small spatial scales (Robertson et al., 1997; Blackwood et al., 2006; Schmidt and Waldron, 2015). For example, analyses done using Illumina next-generation sequencing indicated that soil bacterial communities from organic farms were shaped more by location than by specific treatment effects (Fernandez et al., 2016a). Quantification of some bacterial species related to nitrification and denitrification processes has been possible due to the development of polymerase chain reaction (PCR) assays (Geets et al., 2007). However, little is known about the diversity of species or strains associated with these gene targets or how abundances of these species might be related to the microbial community as a whole.

In this study, we used an Illumina next-generation sequencing approach to characterize the bacterial and archaeal communities in eight soil types with a range of chemical and physical properties collected from research fields distributed across the state of Minnesota. These soils were subsequently amended with varying concentrations of urea, as was described previously (Breuillin-Sessoms et al., 2017). We hypothesized that, despite site-specific differences in bacterial and archaeal communities, urea amendment would have a common effect on the abundance and diversity of members of the microbial community associated with nitrification processes, as well as potentially related taxa, across all soils. Furthermore, we hypothesized that urea amendment would further affect the broader heterotrophic community resulting in decreased diversity and variation in abundances of specific taxa. Results of this study provide novel insights into the response of the total bacterial and archaeal community to urea amendment and describe specific shifts in populations of nitrifiers associated with N cycling.

## Materials and Methods

### Sampling Sites and Soil Characterization

Agricultural soil samples were collected from eight research fields at University of Minnesota Research and Outreach centers distributed throughout the state, as previously described (Breuillin-Sessoms et al., 2017). Sites included Becker, Crookston, Lamberton, Waseca, Rosemount (two sites, tilled and non-tilled) (Venterea et al., 2006), and Saint Paul (two sites, cropped with either corn or soybeans). Samples were collected in 2014 following the autumn crop harvest from treatments receiving no N fertilizer addition. Soils were dried at room temperature for 7–10 days, sieved (2 mm), homogenized, and stored at 4°C until used. Initial soil composition and edaphic parameters are reported in Supplementary Table S1 as previously described (Breuillin-Sessoms et al., 2017).

### Microcosm Design and Chemical Analyses

Microcosm experiments were performed in sterile 250-mL glass microcosm jars as previously described (Breuillin-Sessoms et al., 2017). Briefly, 10–13 g soil from a single sampling site was added to a series of replicated microcosms and the moisture content was adjusted to 85% water holding capacity containing reagent grade urea [CO(NH_2_)_2_] and purified water at treatment levels of 0 (water only), 100, and 500 μg N g^-1^ dry soil. These urea concentrations were selected to cover a range of soil chemical conditions following different N fertilizer application practices, with the greater concentration (500 μg N g^-1^) representing potential conditions within fertilizer bands and/or within close proximity to urea granules (Wetselaar et al., 1972; Yadvinger-Singh and Beauchamp, 1988), whereas the lower (100 μg N g^-1^) concentration is more representative of uniform (broadcast) application methods. Microcosms were maintained under aerobic conditions in the dark at 22°C through a 21-day sample collection. A destructive sampling method was used, with triplicate jars per soil type and urea amendment sacrificed at 0, 4, 14, and 21 days for next-generation sequence analysis. Analyses of several chemical variables were performed as described previously (Breuillin-Sessoms et al., 2017) (summarized here briefly in Supplementary Methods) and values used in the present study are summarized in Supplementary Table S2. Effects of drying/re-wetting soils were not specifically evaluated in this study, although these physical dynamics may also impact changes in the microbial community composition in a soil-specific manner (Fierer et al., 2003).

### DNA Extraction and Quantitative PCR Assays

DNA was extracted from 250 mg (wet weight) of soil using the DNeasy^®^ PowerSoil Kit^®^ (QIAGEN, Hilden, Germany) according to the manufacturer’s instructions, with the final elution performed twice. The DNA extracts were subjected to qPCR analysis of abundances for *amoA*, *nxrA*, and *nxrB* genes that were previously reported (Breuillin-Sessoms et al., 2017). These qPCR data are included in the current study only as metadata to offer some contextualization of 16S rRNA amplicon sequencing results. Quantitative PCR (qPCR) analyses were performed on 1:10 dilutions of DNA extracts in PCR-grade water using the 7500 Fast Real Time PCR system (Applied Biosystems, Foster City, CA, United States) and iTaq Universal SYBR Green Supermix (Bio-Rad Laboratories, Inc., Hercules, CA, United States) as described previously (Breuillin-Sessoms et al., 2017). For enumeration of the 16S rRNA gene, the 515F/806R primer set targeting the V4 hypervariable region was used (Caporaso et al., 2012). For all assays, samples were run in triplicate, with no-template-added negative controls. Standard curves were used to estimate gene copy number, with *r*^2^ values ≥ 0.99 and efficiencies between 80 and 95%. Quantitative PCR data used in the present study are summarized in Supplementary Table S3.

### Next-Generation Sequencing

Undiluted DNA (see above) was also used as a template for amplification and Illumina next-generation sequencing of the V4 hypervariable region using the dual index method by the University of Minnesota Genomics Center [UMGC, (Gohl et al., 2016)]. Briefly, samples were amplified using 25 cycles with primers without full adapter sequences or barcoding indices, followed by addition of adapters and barcodes with an additional 10 cycles of PCR. Amplicons were size-selected, pooled, and paired-end sequenced at a read length of 250 nucleotides (nt) on the Illumina HiSeq2500 platform (Illumina, Inc., San Diego, CA, United States) by UMGC. Sequence data were returned as fastq files and are deposited in the Sequence Read Archive of the National Center for Biotechnology Information under BioProject accession number SRP106784.

### Bioinformatics

Sequence data were processed and analyzed using mothur ver. 1.35.1 (Schloss et al., 2009), and processed as described previously (Staley et al., 2015). Briefly, samples were trimmed to 160 nt, paired-end joined using fastq-join software (Aronesty, 2013), and trimmed for quality based on quality score (>35), homopolymer length (8 nt), ambiguous bases, and primer mismatches (2 nt). Samples were aligned against the SILVA database ver. 123 (Pruesse et al., 2007), subjected to a 2% pre-cluster step to remove sequence errors (Huse et al., 2010), and chimeras were identified and removed using UCHIME (Edgar et al., 2011). Operational taxonomic units (OTUs) were identified at 97% similarity using the complete-linkage algorithm and taxonomic classifications were made using the version 14 release from the Ribosomal Database Project (Cole et al., 2009).

Alpha diversity indices, as well as Good’s coverage, were calculated using the Shannon index and abundance-based coverage estimate (ACE) using mothur. Differences in beta diversity were evaluated using Bray–Curtis dissimilarity matrices (Bray and Curtis, 1957), which accounts for differences in abundance of OTUs. Differences in community composition were evaluated by analysis of similarity (ANOSIM) (Clarke, 1993), while differences in sample clustering were evaluated by analysis of molecular variance (AMOVA) (Excoffier et al., 1992). Ordination of samples was performed by principal coordinate analysis (PCoA) (Anderson and Willis, 2003). Operational taxonomic units that differed significantly in abundance by treatment were determined using linear discriminant analysis of effect sizes (LEfSe) (Segata and Huttenhower, 2011), and were subsequently classified to families. Oligotyping analyses were used to determine potential species- and strain-level distributions among nitrifiers, according to recommended best practices (Eren et al., 2013). An oligotype was defined as present in more than 1% of reads, present in at least three samples, the most abundant unique sequence occurred at a minimum of 50 reads, and if the minimum read abundance throughout the dataset was at least 250.

### Statistical Analyses

For statistical analyses, chemical analyte and qPCR data were log_10_ transformed, as performed previously (Breuillin-Sessoms et al., 2017), and bacterial and archaeal abundances were evaluated as percentages of total sequence reads. For statistical comparisons, all samples were rarefied to 50,000 reads per sample by random subsampling (Gihring et al., 2012). Statistics were calculated using XLSTAT ver. 2015.5.01 (Addinsoft, Belmont, MA, United States). ANOVA analyses were performed with Tukey’s *post hoc* test and correlations were determined using Spearman rank correlations. Canonical correspondence analysis was used to describe multivariate analyses. Variance partitioning was performed using partial redundancy analysis as described previously (Borcard et al., 1992), using the vegan package in R ver. 3.2.2 (R Core Team, 2012; Oksanen et al., 2015). All statistics were evaluated at α = 0.05 with Bonferroni correction for multiple comparisons.

## Results

### Bacterial and Archaeal Community Alpha Diversity

Bacterial and archaeal communities showed high species richness among all samples, with a mean abundance-based coverage estimate (ACE) of 11,179 ± 2,570 (mean ± standard deviation, range: 5,564–20,216). The mean estimated Good’s coverage, after rarefaction, was 95.1 ± 0.8%, among all samples. Samples from Becker, both the Rosemount sites, and the St. Paul (soybean) soil had significantly greater Shannon indices (means 6.94 – 6.96, **Table 1**) than did the Crookston, Lamberton, and St. Paul (corn) soils (means 6.71 – 6.79; Tukey’s *post hoc P* < 0.05). Samples from St. Paul (corn) and Waseca had intermediate Shannon indices among all sites (6.79 ± 0.30 and 6.90 ± 0.10, respectively), while those from Crookston and Lamberton were significantly lower than all the other sites (6.71 ± 0.22 and 6.72 ± 0.09, respectively; *P* < 0.05), except St. Paul (corn).

**Table 1:** Shannon indices (mean ± standard deviation) of microbial communities in triplicate soil samples.

Urea (μg N g^-1^)	Day	Becker	Crookston	Lamberton	Waseca	Rosemount (tilled)	Rosemount (non-tilled)	St. Paul (corn)	St. Paul (soybean)
0	0	7.24 ± 0.19^A^	6.78 ± 0.03^A-K^	6.77 ± 0.08^B-K^	6.94 ± 0.01^A-I^	7.04 ± 0.04^∗,A-F^	7.09 ± 0.05^A-D^	7.06 ± 0.03^A-F^	7.09 ± 0.04^A-E^
0	4	6.97 ± 0.21^A-I^	6.83 ± 0.07^A-K^	6.65 ± 0.07^C-K^	6.94 ± 0.01^ABC^	7.01 ± 0.04^A-F^	6.96 ± 0.05^A-I^	6.94 ± 0.08^A-I^	6.98 ± 0.05^A-I^
0	14	7.22 ± 0.06^AB^	6.80 ± 0.06^A-K^	6.85 ± 0.10^A-K^	6.93^†,A-F^	6.99 ± 0.05^A-H^	6.98 ± 0.07^A-I^	7.01 ± 0.00^A-F^	6.97 ± 0.02^A-I^
0	21	7.14 ± 0.01^A-I^	6.80 ± 0.01^C-K^	6.78 ± 0.07^A-K^	6.87 ± 0.04^∗,A-I^	7.07 ± 0.06^A-F^	6.97 ± 0.05^A-F^	6.93 ± 0.04^B-K^	6.99 ± 0.03^A-F^
100	4	7.00 ± 0.05^A-H^	6.80 ± 0.01^A-K^	6.69 ± 0.03^C-K^	7.04 ± 0.10^A-F^	6.89 ± 0.04^A-K^	6.90 ± 0.08^A-J^	6.89 ± 0.01^A-K^	6.95 ± 0.03^A-I^
100	14	7.03 ± 0.11^A-F^	6.85 ± 0.01^A-K^	6.69 ± 0.03^C-K^	6.87 ± 0.01^A-I^	6.98 ± 0.02^A-I^	6.98 ± 0.03^A-I^	6.91 ± 0.02^A-J^	6.95 ± 0.04^∗,A-I^
100	21	7.09 ± 0.06^A-I^	6.77 ± 0.05^C-K^	6.76 ± 0.08^A-K^	6.94 ± 0.17^A-K^	7.00 ± 0.06^A-G^	6.91 ± 0.04^A-F^	6.88 ± 0.01^C-K^	6.96 ± 0.05^A-F^
500	4	6.63 ± 0.07^A-K^	6.78 ± 0.04^F-K^	6.63 ± 0.04^C-K^	6.87 ± 0.01^A-K^	6.92 ± 0.03^A-J^	6.97 ± 0.08^A-I^	6.88 ± 0.09^D-K^	6.96 ± 0.02^A-I^
500	14	6.85 ± 0.14^B-K^	6.55 ± 0.02^IJK^	6.69 ± 0.06^E-K^	6.81 ± 0.05^C-K^	6.86 ± 0.02^A-K^	6.89 ± 0.09^A-K^	6.23 ± 0.08^G-K^	6.92 ± 0.05^A-K^
500	21	6.46 ± 0.11^C-K^	6.10 ± 0.02^K^	6.67 ± 0.07^H-K^	6.77 ± 0.09^F-K^	6.65 ± 0.30^C-K^	6.88 ± 0.05^B-K^	6.20 ± 0.07^JK^	6.86 ± 0.06^B-K^


**^*A-K*^Samples sharing the same superscript did not differ significantly by Tukey’s post hoc test (P > 0.05). ^∗^Only two samples returned sufficient sequence reads for analysis. ^†^Only one sample returned sufficient sequence reads for analysis.**

Variation in alpha diversity resulting from urea amendment and time post-treatment showed soil-specific patterns (**Table 1**). Samples from soils amended with 500 μg N g^-1^ soil (6.71 ± 0.25) had significantly lower Shannon indices than unamended or 100-μg-N-treated soils (*P* < 0.0001). The most significant shifts in Shannon indices (e.g., in the 500 μg N g^-1^ treatment at Becker on day 21) represented a loss of up to 2,800 OTUs following treatment, as estimated by ACE richness. At the 14 and 21 days post-treatment (6.86 ± 0.20 and 6.81 ± 0.26, respectively) experimental conditions resulted in significantly lower Shannon diversity than that observed prior to any treatment (7.00 ± 0.17; *P* ≤ 0.037). In contrast, there were no differences in Shannon indices in samples collected from unamended soil or soil amended with 100 μg N g^-1^ soil (means 6.96 ± 0.14 and 6.90 ± 0.12, respectively; *P* = 0.259), among all sites.

### Bacterial and Archaeal Community Composition and Beta Diversity

Bacterial communities among all soils were primarily comprised of members of the bacterial phyla *Actinobacteria, Proteobacteria*, and *Acidobacteria*, which together represented more than 65% of sequence reads in all samples and time points (Supplementary Figure S1). Several sites also showed abundances of members of the archaeal phylum *Thaumarchaeota* that represented up to 13% of sequence reads. When classified to families (Supplementary Figure S2), members of the *Gaiellaceae* and *Sphingomonadaceae* were among the predominant bacteria classified, while nearly all archaeal taxa were classified within the genus *Nitrososphaera* in the family *Nitrososphaeraceae*. Taxa representing approximately 20–30% of sequence reads could not be further classified to bacterial and archaeal families, and members of low abundance families (consolidated in Supplementary Figure S1 and found at mean abundances < 1.5% of sequence reads) accounted for another 30–40% of sequence reads.

**FIGURE 1:**
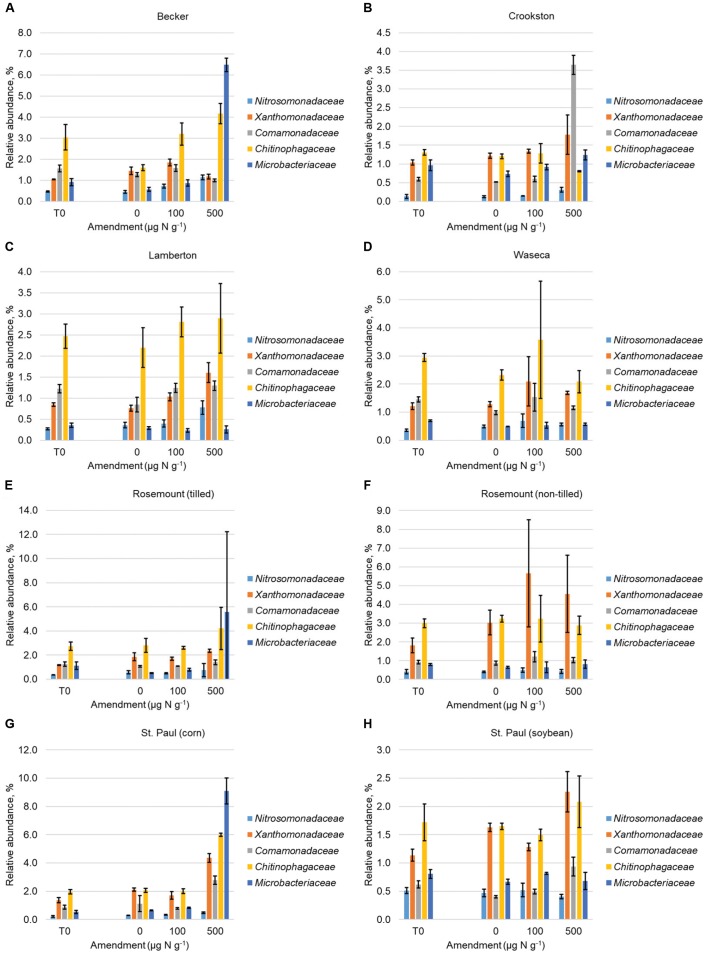
Abundances (mean ± standard deviation) of families to which OTUs that were discriminatory for urea amendment (Supplementary Figure S3) were classified. Abundances are shown prior to urea amendment and at the 21-day time point for each amendment at **(A)** Becker, **(B)** Crookston, **(C)** Lamberton, **(D)** Waseca, **(E)** Rosemount (tilled), **(F)** Rosemount (non-tilled), **(G)** St. Paul (corn), **(H)** St. Paul (soybean).

Linear discriminant analysis (LDA) of effect sizes (LEfSe) showed that OTUs classified within the families *Microbacteriaceae*, *Chitinophagaceae*, *Comamonadaceae*, *Xanthomonadaceae*, and *Nitrosomonadaceae* were predominantly identified as taxa with greater abundances in soils receiving more concentrated urea amendments (LDA score ≥ 3.0), in individual soils among all time points (data not shown). When LEfSe analysis was applied to all samples and time points, only five OTUs were identified as having greater abundance in the 500 μg N g^-1^ soil amendment, and the OTUs were similarly classified to these same families (Supplementary Figure S3). Changes in abundance of these families reflected an increase at greater urea amendment through the 21-day time point (**Figure 1**), although we did observe site-specific differences in the magnitude of shifts among families. When all samples were analyzed to determine consistent variation by time, only two OTUs within the *Xanthomonadaceae* were identified as indicators of the 4- and 21-day time points (one OTU per time point).

**FIGURE 2:**
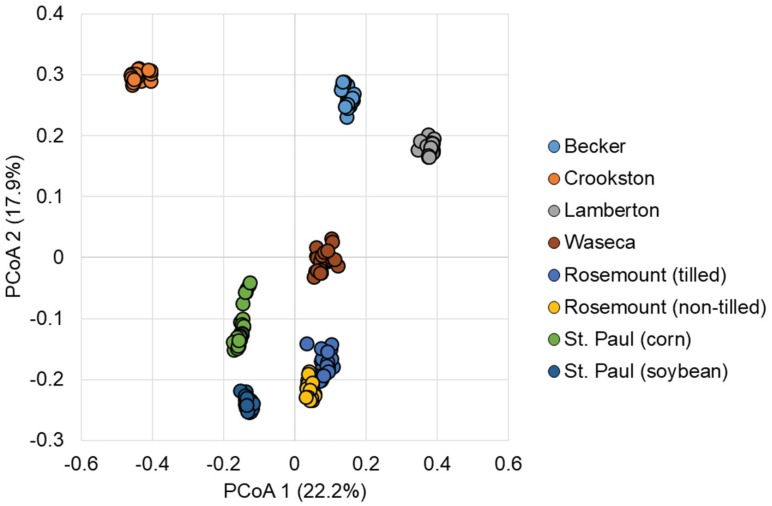
Principal coordinate analysis of Bray–Curtis dissimilarity matrices among all samples (*r*^2^ = 0.76).

Ordination of Bray–Curtis dissimilarity matrices (a measure of difference in abundances of OTUs) by principal coordinate analysis (PCoA, **Figure 2**) revealed independent clustering of samples by soil, as evaluated by analysis of molecular variance (AMOVA, *P* < 0.001). Grouping of samples based on urea treatment or time-post-treatment were not significant (*P* = 0.110 and 0.744, respectively), among all samples. These groupings were also supported by differences in community composition, evaluated by analysis of similarity (ANOSIM, *P* < 0.001 for pairwise soil comparisons and *P* = 0.078 and 0.595 for treatment and time, respectively).

Within a site-specific soil type (Supplementary Figure S4), significant temporal shifts in community composition were generally observed between the 4-day time point and those taken 14 or 21 days following treatment (*P* < 0.008), although significant temporal shifts were not observed in the Waseca or Rosemount (tilled) soils. However, urea amendments had more significant effects than did temporal variation on community composition (ANOSIM *R* = 0.32 ± 0.14 and 0.22 ± 0.07, respectively; **Table 2**).

**Table 2:** Beta diversity statistics relating communities by urea treatment at each sampling site.

Site	Urea add (μg N/g soil)	0	100	500
**Becker**	**0**		**0.35, 0.003**	**0.85, <0.001**
	**100**	**2.48, 0.004**		**0.74, <0.001**
	**500**	**8.36, <0.001**	**6.47, <0.001**	
**Crookston**	**0**		<-0.01, 0.44	**0.40, 0.001**
	**100**	1.23, 0.051		**0.29, 0.007**
	**500**	**4.71, <0.001**	**3.93, 0.004**	
**Lamberton**	**0**		0.07, 0.118	**0.25, 0.003**
	**100**	1.28, 0.106		0.16, 0.020
	**500**	**2.40, 0.006**	**1.94, 0.004**	
**Waseca**	**0**		**0.35, 0.005**	**0.25, 0.001**
	**100**	**3.62, 0.005**		0.12, 0.082
	**500**	1.96, 0.068	1.35, 0.198	
**Rosemount (tilled)**	**0**		**0.33, 0.002**	**0.42, <0.001**
	**100**	**2.83, 0.001**		**0.25, <0.001**
	**500**	**3.78, <0.001**	**2.25, <0.001**	
**Rosemount (non-tilled)**	**0**		**0.20, 0.008**	**0.60, <0.001**
	**100**	**1.92, 0.004**		0.10, 0.048
	**500**	**3.61, <0.001**	1.42, 0.075	
**St. Paul (corn)**	**0**		0.05, 0.167	**0.64, <0.001**
	**100**	**1.55, 0.015**		**0.56, <0.001**
	**500**	**8.03, <0.001**	**6.91, <0.001**	
**St. Paul (soybean)**	**0**		0.20, 0.026	**0.56, <0.001**
	**100**	**1.53, 0.006**		**0.23, 0.008**
	**500**	**3.13, <0.001**	1.77, 0.033	


*ANOSIM R-values and P-values are presented in the top matrix for each soil type. AMOVA F-statistic and P-values are presented in the bottom matrix for each soil type. Significance values are shown in bold. All statistics were evaluated at Bonferroni-corrected α = 0.02.*

### Variation in Edaphic Parameters, Community Composition, and Nitrification Genes

Variance partitioning by partial redundancy analysis was used to evaluate drivers of community variation at the 21-day time point. This was taken as the relative abundances of all bacterial and archaeal families (*n* = 348) that was attributable to initial edaphic parameters prior to amendment (Supplementary Table S1), soil chemical properties following treatment (Supplementary Table S2), along with urea amendment, or both initial and treatment effects. The initial edaphic properties, including soil composition, pH, organic matter, organic N, organic C, cation exchange capacity, K, and moisture content accounted for 17.7% of community variation, among all soils. Furthermore, only 22.0% of variation was explained by concentrations of N-chemical analytes, H, CO_2_, and urea. The remaining 60.3% of variation was constrained by these variables interacting in some combination to influence the bacterial and archaeal communities.

**FIGURE 3:**
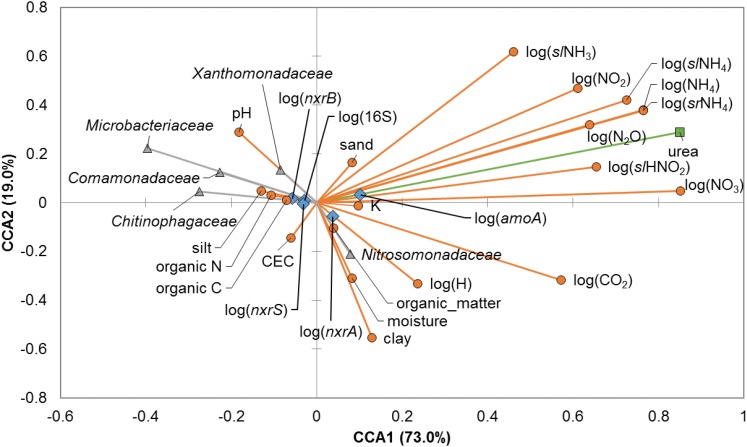
Canonical correspondence analysis (CCA) relating bacterial families, edaphic and physicochemical parameters, and abundances of nitrification genes at 21 days post-treatment. Bacterial families presented are those in which OTUs determined to differ significantly due to nitrogen treatment by LEfSe analysis were classified. Relative abundances of all members of the families were used for CCA. Edaphic parameters and N-chemical analytes are shown as orange circles, abundances of bacterial families are shown as gray triangles, urea amendment is shown as a green square, and nitrification genes are shown as blue diamonds.

Canonical correspondence analysis (**Figure 3**) showed strong and significant correlations between the urea amendment concentrations and N-analytes or CO_2_ at 21 days post-treatment (Spearman’s ρ = 0.363 – 0.886, *P* < 0.05), and the majority of N-analytes were significantly and positively correlated amongst each other. Urea amendment concentration was also positively correlated with gene abundances of bacterial *amoA* and *nxrA* (ρ = 0.923 and 0.513, *P* < 0.0001). Conversely, the relative abundances of all members of the *Microbacteriaceae*, *Chitinophagaceae*, *Comamonadaceae*, and *Xanthomonadaceae* were significantly negatively correlated with the concentrations of many N-analytes, including N_2_O, and gene abundances of *amoA* and *nxrA*. Soil pH was significantly and positively correlated with abundances of *Microbacteriaceae* (ρ = 0.679, *P* < 0.0001), but was negatively correlated with abundances of the *Chitinophagaceae, Comamonadaceae*, and *Nitrosomonadaceae* (ρ = -0.561 to -0.394, *P* ≤ 0.001). Other soil edaphic parameters tended to be positively inter-correlated with fewer significant relationships to N-analytes, relative abundances of bacterial families, or nitrification gene abundances.

### Oligotyping of Nitrifying Prokaryotes

**FIGURE 4:**
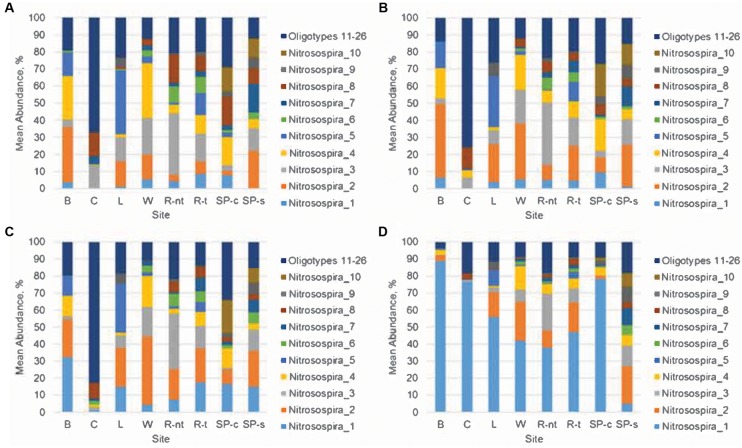
Distribution of oligotypes within the genus *Nitrosospira*. **(A)** Initial sampling, prior to treatment, **(B)** no urea amendment, day 21, **(C)** 100 μg N g^-1^ soil amendment, day 21, **(D)** 500 μg N g^-1^ soil amendment, day 21. Sampling sites include Becker (B); Crookston (C); Lamberton (L); Waseca (W); Rosemount (non-tilled, R-nt); Rosemount (tilled, R-t), St. Paul (corn, SP-c), and St. Paul (soybean, SP-s).

**FIGURE 5:**
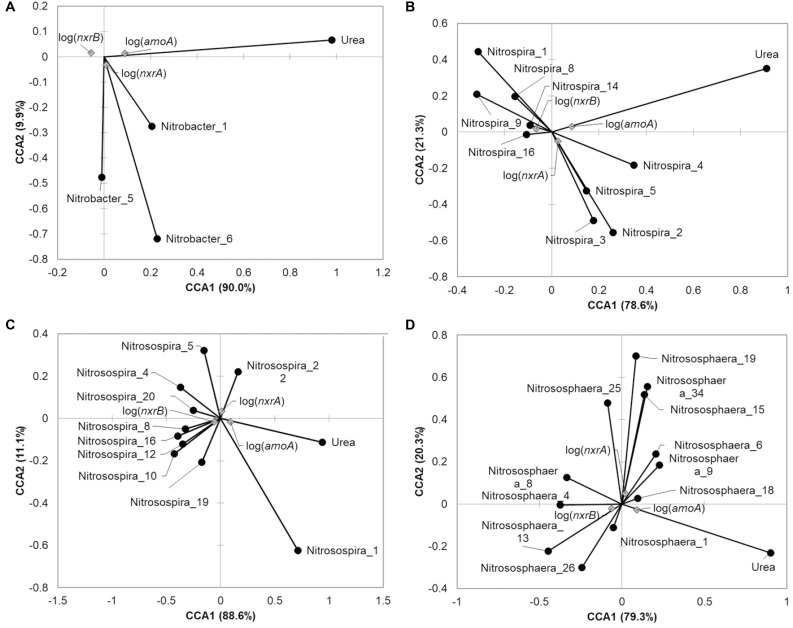
Canonical correspondence analysis relating oligotypes and urea amendment concentration to abundance of nitrification genes at 21 days post-treatment. Relative abundance of the oligotype within the genus was used for analysis for **(A)** *Nitrobacter*, **(B)** *Nitrospira*, **(C)** *Nitrosospira*, or **(D)** *Nitrososphaera.* Only those oligotypes that showed significant Spearman correlations (*P* < 0.05) with nitrification genes are shown.

Oligotyping analyses were done to determine the distribution of likely species or strains among the nitrifying genera *Nitrosospira, Nitrospira*, *Nitrobacter*, and *Nitrososphaera* (**Table 3**, **Figure 4**, and Supplementary Figures S5–S7), as well as their relationships to urea amendment and nitrification gene abundances (**Figure 5**). The distributions of oligotypes within the bacterial genera showed considerably greater, soil-specific shifts due to urea amendment concentration through the 21-day time point (**Figure 4** and Supplementary Figures S5–S7), relative to the archaeal genus *Nitrososphaera* (Supplementary Figure S7). Importantly, the relative abundances of only two oligotypes within the *Nitrobacter* or *Nitrospira* were significantly (*P* < 0.05) correlated with urea amendment concentration. In both genera, one oligotype was positively correlated, while the other was negatively correlated with amendment concentration. Similarly, among *Nitrosospira*, only the abundance of one oligotype was significantly positively correlated with urea concentration, while four were negatively correlated. Oligotyping was not performed on *Nitrosomonas* due to a low number of OTUs classified (*n* = 7, compared to >100 for other genera oligotyped).

**Table 3:** Mean abundances (±standard deviation, as %) of nitrifying prokaryotic taxa interrogated by oligotyping.

Site	Nitrosospira	Nitrospira	Nitrobacter	Nitrososphaera
Becker	0.61 ± 0.27 (0.20 - 1.27)	0.39 ± 0.17 (0.13 - 0.67)	0.10 ± 0.04 (0.05 - 0.25)	1.48 ± 0.51 (0.67 - 2.89)
Crookston	0.21 ± 0.14 (0.07 - 0.74)	0.48 ± 0.09 (0.27 - 0.66)	0.14 ± 0.03 (0.07 - 0.21)	12.18 ± 1.16 (10.05 - 14.49)
Lamberton	0.50 ± 0.26 (0.25 - 1.42)	0.32 ± 0.06 (0.21 - 0.48)	0.16 ± 0.03 (0.11 - 0.22)	3.97 ± 0.94 (1.68 - 5.82)
Waseca	0.67 ± 0.26 (0.32 - 1.34)	0.32 ± 0.07 (0.19 - 0.43)	0.07 ± 0.02 (0.04 - 0.11)	4.86 ± 1.64 (1.83 - 7.17)
Rosemount (tilled)	0.58 ± 0.25 (0.21 - 1.28)	0.43 ± 0.11 (0.20 - 0.57)	0.09 ± 0.03 (0.04 - 0.15)	6.85 ± 1.47 (3.29 - 9.74)
Rosemount (non-tilled)	0.41 ± 0.09 (0.26 - 0.63)	0.29 ± 0.07 (0.14 - 0.46)	0.14 ± 0.03 (0.09 - 0.20)	4.87 ± 1.11 (2.45 - 8.51)
St. Paul (corn)	0.36 ± 0.11 (0.16 - 0.58)	0.42 ± 0.10 (0.20 - 0.56)	0.02 ± 0.01 (0.00 - 0.05)	5.13 ± 1.45 (2.19 - 7.08)
St. Paul (soybean)	0.56 ± 0.25 (0.36 - 1.55)	0.50 ± 0.07 (0.35 - 0.64)	0.32 ± 0.42 (0.07 - 1.26)	5.41 ± 0.74 (3.05 - 6.41)


**Range is shown in parentheses. All samples from each site are summarized, regardless of time point or treatment*.*

The *Nitrobacter* oligotypes that were significantly related to nitrification gene abundances tended also to be positively correlated with abundances of *nxrA* and negatively correlated with *nxrB*. The oligotype 1 was also significantly positively correlated with bacterial *amoA* abundances (**Figure 5A**). Conversely, while several of the *Nitrospira* were positively correlated with abundances of *nxrB*, others showed positive correlations with *nxrA*, but only one (oligotype 1) showed a significant negative correlation with *amoA* (**Figure 5B**). Among the *Nitrosospira*, oligotype 1 was significantly positively correlated with *amoA* while other oligotypes that showed significant correlations with nitrification genes were positively correlated with *nxrB* (**Figure 5C**). Among the *Nitrososphaera* (**Figure 5D**), significant positive correlations were observed with oligotype abundances and *nxrA* or *nxrB*, but few showed significant relationships with more than one gene target.

## Discussion

Bacterial and archaeal communities characterized in this study primarily showed variation as a result of sample location, similar to previous findings using Illumina sequencing in organic agricultural soils (Fernandez et al., 2016a,b). Soil-specific communities were further supported by variance partitioning and the initial soil characteristics explained 18% of the community, consistent with previous studies that demonstrated legacy effects of moisture on both soil community composition and N_2_O flux (Banerjee et al., 2016). Among all sites and soils, the amendment with 500 μg N g^-1^ resulted in declines in alpha diversity and consistent expansions in abundances of members of the families *Microbacteriaceae*, *Chitinophagaceae*, *Comamonadaceae*, *Xanthomonadaceae*, and *Nitrosomonadaceae*, typically by 14 days following amendment. The rapidity and extent of these changes, however, varied in a soil-dependent manner. In contrast, amendment with 100 μg N g^-1^ soil did not greatly affect overall alpha diversity in soils, but did result in significant community shifts in most of the tested soils that could not be attributed to specific taxa. This decrease in diversity is consistent with meta-analyses showing decreased microbial diversity in response to urea, but not other mineral fertilizers (Geisseler and Scow, 2014). Results of our study align well with the previous postulated subsidy-stress hypothesis, where low levels of urea amendment may stimulate N cycling in the communities, by either increasing microbial diversity or activity. In contrast, greater levels prove toxic to members of the microbial community resulting in decreased diversity (Odum et al., 1979; Odum, 1985). Similarly, in rice paddy soils amended with 200 μg N g^-1^ soil, urea amendment did not significantly affect alpha diversity, but also had no significant impact on community composition (Wang et al., 2015), although a more shallow sequencing depth (∼13,000 reads) was used in the previous study.

We hypothesized that urea amendments would result in consistent shifts in the bacterial and archaeal soil communities, despite high diversity and soil-specific variation in community composition and edaphic parameters, potentially due to a direct effect of this chemical amendment or changes in physicochemical parameters associated with reduced heterotrophic respiration (Janssens et al., 2010; Ramirez et al., 2010). Among all the soils examined, OTUs that classified to five families (*Microbacteriaceae*, *Chitinophagaceae*, *Comamonadaceae*, *Xanthomonadaceae*, and *Nitrosomonadaceae*) were consistently found to be indicators of greater urea amendment concentrations. Members of these families have recently been reported to be highly ubiquitous and relatively abundant in soils globally (Delgado-Baquerizo et al., 2018). Increases in their relative abundances may reflect some resistance to changes in soil chemistry as a result of urea application (e.g., increased pH and NH_3_ concentrations) that are otherwise inhibitory to microorganisms (Geisseler and Scow, 2014). Further research will be required to determine if these families are associated with the decline in heterotrophic respiration that is typically observed (Janssens et al., 2010). Although a previous study did not find significant differences in rice paddy bacterial communities associated with urea addition (Wang et al., 2015), another noted an increase in AOB following short-term urea amendment in an alpine grassland (Xiang et al., 2017). Similarly, we previously observed a positive correlation between bacterial *amoA* abundances and N_2_O (Pearson’s *r* = 0.62) and NO_2_^-^ (*r* = 0.66) (Breuillin-Sessoms et al., 2017). In contrast, AOB were found to respond to inorganic N, while AOA abundances increased in response to organic N following chronic application in clay loam soils, and this suggested that soil type and temporal dynamics may affect shifts in communities (Zhou et al., 2015). Taken together, these results suggest that the decline in abundance of these families is likely associated with more general species sorting dynamics (e.g., changes in pH and/or nutrient availability) rather than specific chemical toxicity, among all conditions tested.

Taxa within the family *Nitrosomonadaceae*, specifically the genus *Nitrosospira*, were also observed in this study to be responsive to urea amendment, although the relative abundance of this family showed weak and insignificant correlations with both urea amendment rate and N_2_O production (ρ = -0.054 and 0.031). Since the microbial community composition was too diverse among all soils to resolve many specific trends related to taxa abundances, we performed oligotyping on the predominant nitrifiers found in the dataset. Oligotyping analyses of nitrifiers revealed diverse oligotypes (species or strains) within the genera *Nitrobacter*, *Nitrospira*, *Nitrosospira*, and *Nitrososphaera*. Typically, only one oligotype within each genus was strongly correlated with nitrification gene abundances, suggesting that only one or a few strains within nitrifier families plays an active role in response to N fertilization. Furthermore, these strain level differences in relation to nitrification genes may explain why taxonomic resolution at the family level did not reveal more discrete trends, in part due to the high diversity of potential nitrifiers (Hayatsu et al., 2008). Future work will be necessary to characterize these specific strains to evaluate their roles in N cycling.

While correlation analyses typically revealed the expected associations between abundances of gene targets and genus-specific oligotype (e.g., positive correlation between *Nitrobacter* and *nxrA* abundances), the implications of potentially greater strain-specific proficiency in carrying out processes of nitrification remains unknown. This finding may reflect a coincidental spatial benefit allowing more efficient cross-talk between AOB and NOB (Mobarry et al., 1996), or could reflect differences in competitive ability of certain strains (D’Costa et al., 2006). Similar to previous findings (Di et al., 2009; Giguere et al., 2017), abundances of *Nitrososphaera* showed considerably less variation due to urea amendment, which may suggest that the contribution of AOA to NH_3_ oxidation in these agricultural soils is less than that of AOB. Stable populations within the *Nitrososphaera*, however, may also reflect a stable and consistent contribution of AOA to nitrification, although exhaustive determination of specific functional shifts was beyond the scope of the current work. Future research will be necessary to assess the contribution of AOA in these soils, since *amoA* of archaea was not measured in this study. Nevertheless, several communities were comprised of relatively large fractions (up to 13%) of *Nitrososphaera*, suggesting that AOA may play a more prominent role in some soils.

Nitrifying bacteria were found to be the dominant participants in N-metabolism, based on relationships with gene abundances, which is most likely due to the oxic conditions in which the study was carried out. Under more anoxic conditions, such as incidences of water inundation or at deeper depths, it is likely that denitrification plays a more significant role (Wang et al., 2015). As described previously (Breuillin-Sessoms et al., 2017), abundances of bacterial *amoA* were moderately to strongly correlated with N_2_O, NO_2_^-^, and NH_3_. The abundances of *nxrA* showed weaker correlations with N_2_O and NO_2_^-^, but not with NH_3_. This likely suggests that NH_3_ toxicity had a stronger inhibitory effect on NOB than AOB, as was previously suggested (Venterea et al., 2015). Despite significant declines in alpha diversity and community composition, few other taxa that were unaffiliated with nitrification and denitrification processes showed consistent directional responses to urea amendment, suggesting that total community composition minimally impacted N-cycling. However, soil-specific, significant shifts in community composition in response to greater concentrations of urea may have important longer-term implications in overall soil productivity and pathogen resistance (Bailey and Lazarovits, 2003; Otto-Hanson et al., 2013). These effects cannot be addressed in the present study due to the relatively short period of sample collection and *in vitro* nature of the present work.

In this study, we found that site-specific dynamics more strongly shape bacterial and archaeal communities than did urea addition, despite microscale variability among soil communities (Robertson et al., 1997; Blackwood et al., 2006). Urea addition, however, also had significant, but soil-specific, impacts on bacterial and archaeal community composition, although consistent shifts in abundances of *Microbacteriaceae*, *Chitinophagaceae*, *Comamonadaceae*, *Xanthomonadaceae*, and *Nitrosomonadacea* were observed. In the soils tested, bacterial genes associated with nitrification were found to be strongly associated with nitrogen intermediates, suggesting that nitrifiers were predominantly active in N-cycling under the oxic conditions used in this experiment. Oligotyping of the abundant nitrifiers revealed potentially strain-level differences in correlations with nitrification gene abundances and soil-specific variation in the relative abundances of oligotypes in response to urea application. These results provide novel insights regarding the shift in bacterial and archaeal community structure in response to urea application, and reveal previously unreported diversity among nitrifiers as well as strain selectivity in response to urea amendment within predominant nitrifier genera in various agricultural soils.

## Author Contributions

CS performed all data analysis and drafted the manuscript. FB-S, RV, and MS conceived of the study design. FB-S performed the sampling and chemical analyses. PW and FB-S performed the qPCR assays. TK assisted with the sequence data processing and analysis. All authors read, edited, and approved the final version of the manuscript.

## Conflict of Interest Statement

The authors declare that the research was conducted in the absence of any commercial or financial relationships that could be construed as a potential conflict of interest.

## Footnotes

1 http://www.fao.org/faostat/en/#data/RF
