# Supplementary material for: Urea Amendment Decreases Microbial Diversity and Selects for Specific Nitrifying Strains in Eight Contrasting Agricultural Soils
Source: Front Microbiol. 2018 Apr 4;9:634. doi: 10.3389/fmicb.2018.00634 (PMC5893814; doi:10.3389/fmicb.2018.00634)
Supplement: Supplementary file 1 [file Presentation_1.PDF]

Urea Amendment Decreases Microbial Diversity and Selects for Specific Nitrifying Strains in Eight Contrasting Agricultural Soils

Supplemental Methods, Tables, and Figures

Christopher Staley[1], Florence Breuillin-Sessoms[1], Ping Wang[1], Thomas Kaiser[1], Rodney T. Venterea[2,3], and Michael J. Sadowsky[1,2,4#]

[1]BioTechnology Institute, University of Minnesota, St. Paul, Minnesota, USA

[2]Department of Soil, Water, and Climate, University of Minnesota, St. Paul, Minnesota, USA

[3]USDA-ARS, Soil & Water Management Research Unit, St. Paul, Minnesota, USA

[4]Department of Plant and Microbial Biology, University of Minnesota, St. Paul, Minnesota, USA

[#]Correspondence: Michael J. Sadowsky, BioTechnology Institute, University of Minnesota, 140 Gortner Lab, 1479 Gortner Ave, Saint Paul, MN 55108; Phone: (612)-624-2706, Email: sadowsky@umn.edu

Running title: Bacterial and archaeal community associated with urea amendment

Chemical analysis methods

Prior to sampling, jars were opened for 5 min to allow equilibration of the headspace with lab air and then sealed. Jar headspace was sampled after 0, 30 and 60 min using a 10-mL polypropylene syringe inserted through rubber septa, and gas samples were transferred to glass vials that were analyzed within 3 d using a gas chromatograph (model 5890, Agilent/Hewlett-Packard) equipped with a Porapak Q column, an electron capture detector and interfaced to an autosampler (model 7000, Teledyne Tekmar). The N_2O production rate was calculated from the increase in N_2O concentration, headspace volume and dry soil mass. The jar contents were split into four subsamples. One (~5 g) subsample was mixed with 38 mL 2 M KCl (pH = 12), shaken for 10 min and filtered for analysis of NO_2^- + NO_3^- and NO_2^- using the Greiss–Ilosvay method with and without Cd reduction, respectively, in the same extract (Mulvaney, 1996; Stevens and Laughlin, 1995) using flow-through injection (Lachat, Loveland, CO) within 24 h of sampling. Concentrations of NO_3^- were calculated by difference. A second (~ 2 g) subsample was mixed with 1 M KCl for pH analysis, and results were converted to H^+ (10^{-pH}). Concentrations of undissociated nitrous acid (HNO_2) were derived from measured pH and NO_2^- using the pKa of HNO_2 (Venterea and Rolston, 2000). A third (~4 g) subsample was mixed with 38 mL 2 M KCl (pH = 5.6), shaken for 1 h and filtered for analysis of extractable ammonium (NH_4^+). Extracts for NH_4^+ were stored at 4ºC and analyzed using the sodium salicylate-nitroprusside method and flow-through injection (Mulvaney, 1996) within 7 d. A fourth (~1 g) subsample was transferred to a plastic vial and stored at -80°C for DNA extraction. The NH_4^+ sorption capacity was determined for each soil using isotherm methods (Venterea et al., 2015), and the resulting sorption parameters were used together with measured NH_4^+ and pH to calculate corresponding solution-phase NH_3 concentrations per Venterea et al. (2015).

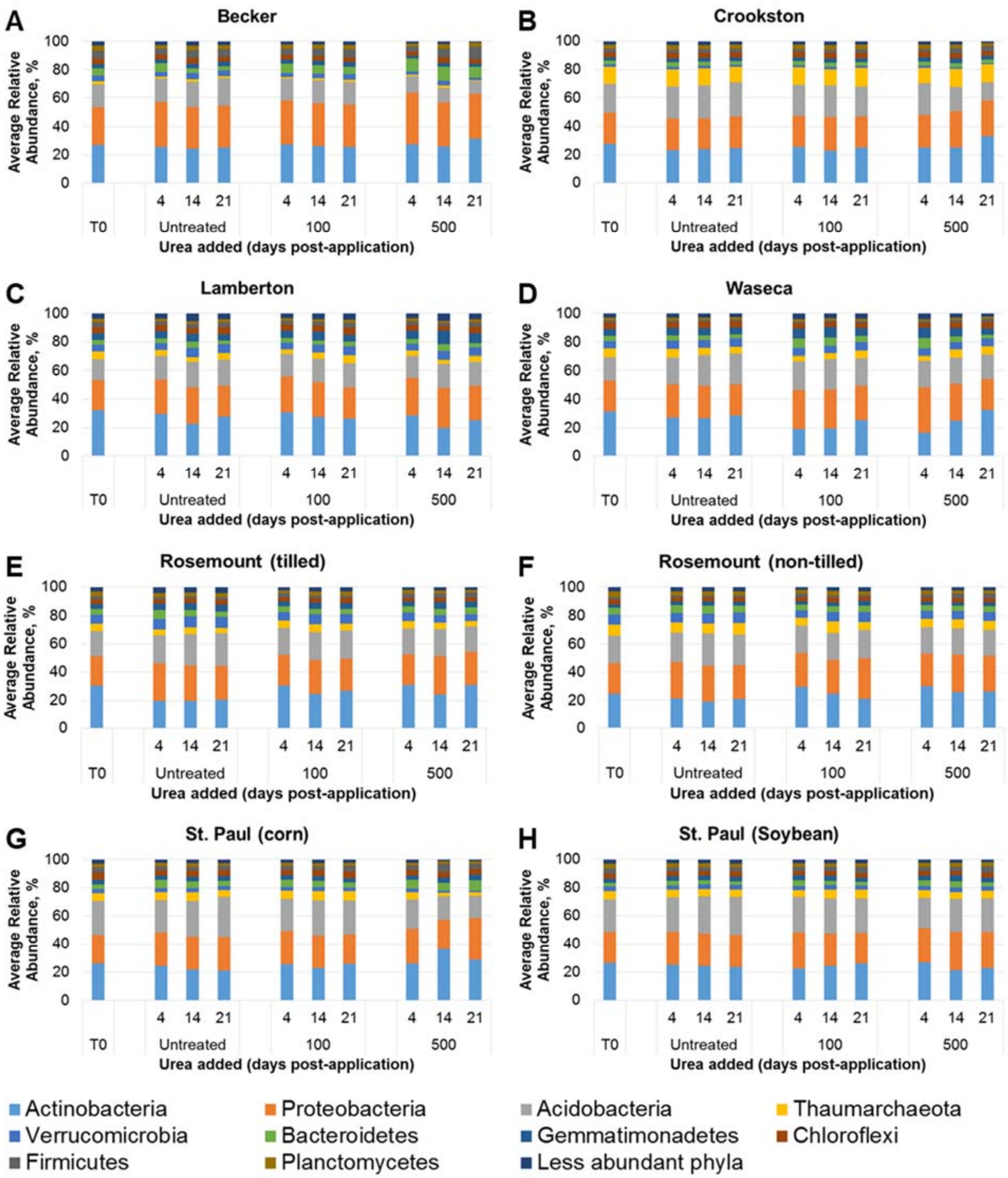

Figure S1 – Distribution of phyla among all samples. A) Becker, B) Crookston, C) Lamberton, D) Waseca, E) Rosemount (tilled), F) Rosemount (non-tilled), G) St. Paul (corn), H) St. Paul (soybean). Urea was added in units of μg N g^{-1} soil.

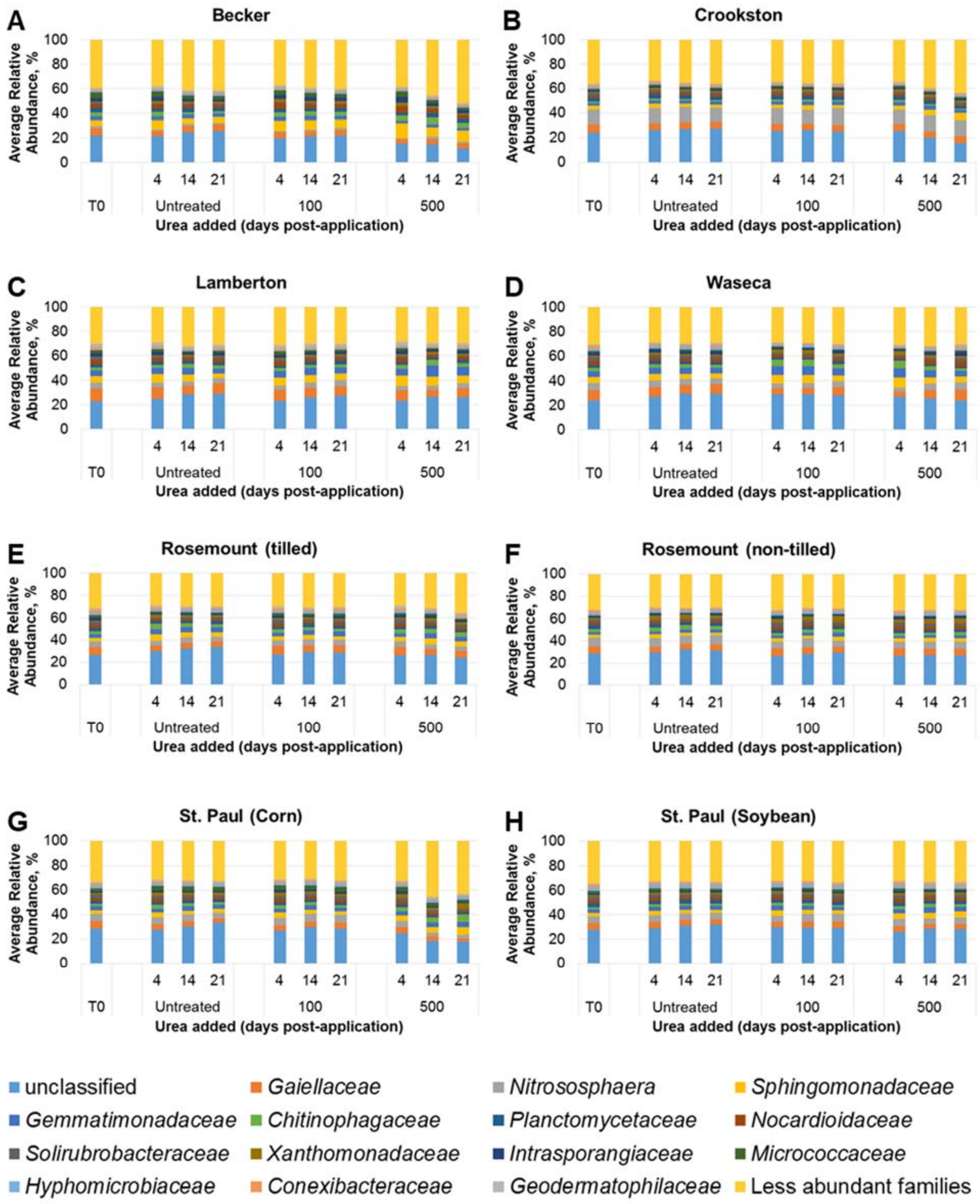

Figure S2 – Distribution of families among all samples. A) Becker, B) Crookston, C) Lamberton, D) Waseca, E) Rosemount (tilled), F) Rosemount (non-tilled), G) St. Paul (corn), H) St. Paul (soybean). Urea was added in units of μg N g^{-1} soil.

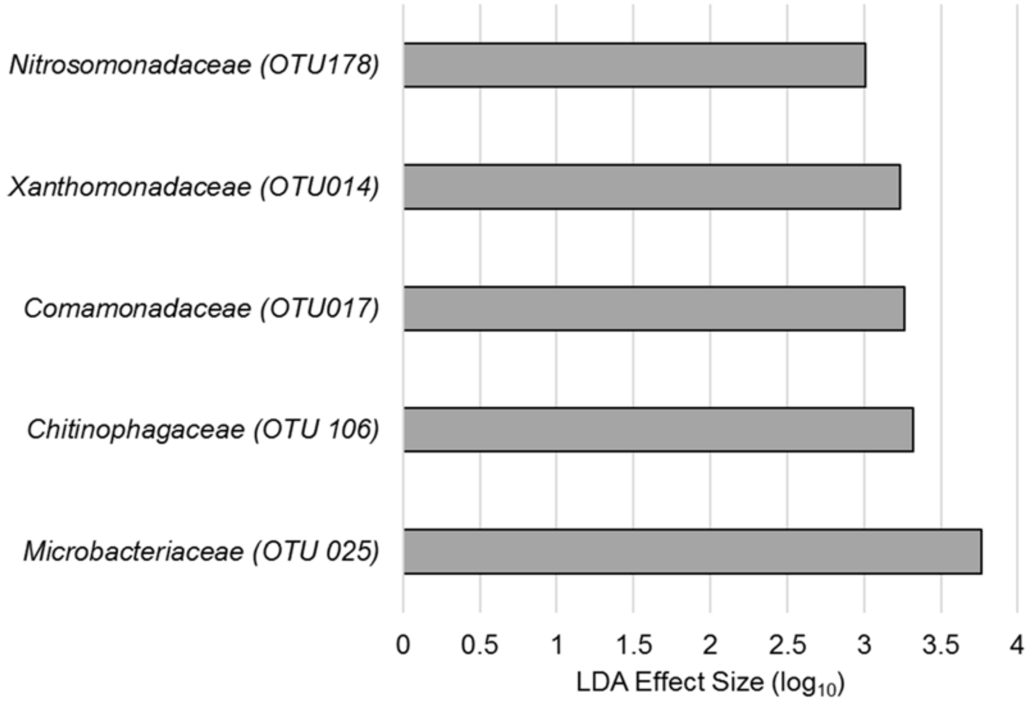

Figure S3 – LEfSe analysis of OTUs, classified to families, that varied significantly as a result of urea treatments among all sites. OTUs were classified to family and all were indicators of the 500 µg N g^{-1} soil treatment. No OTUs were identified as significant markers for other treatments.

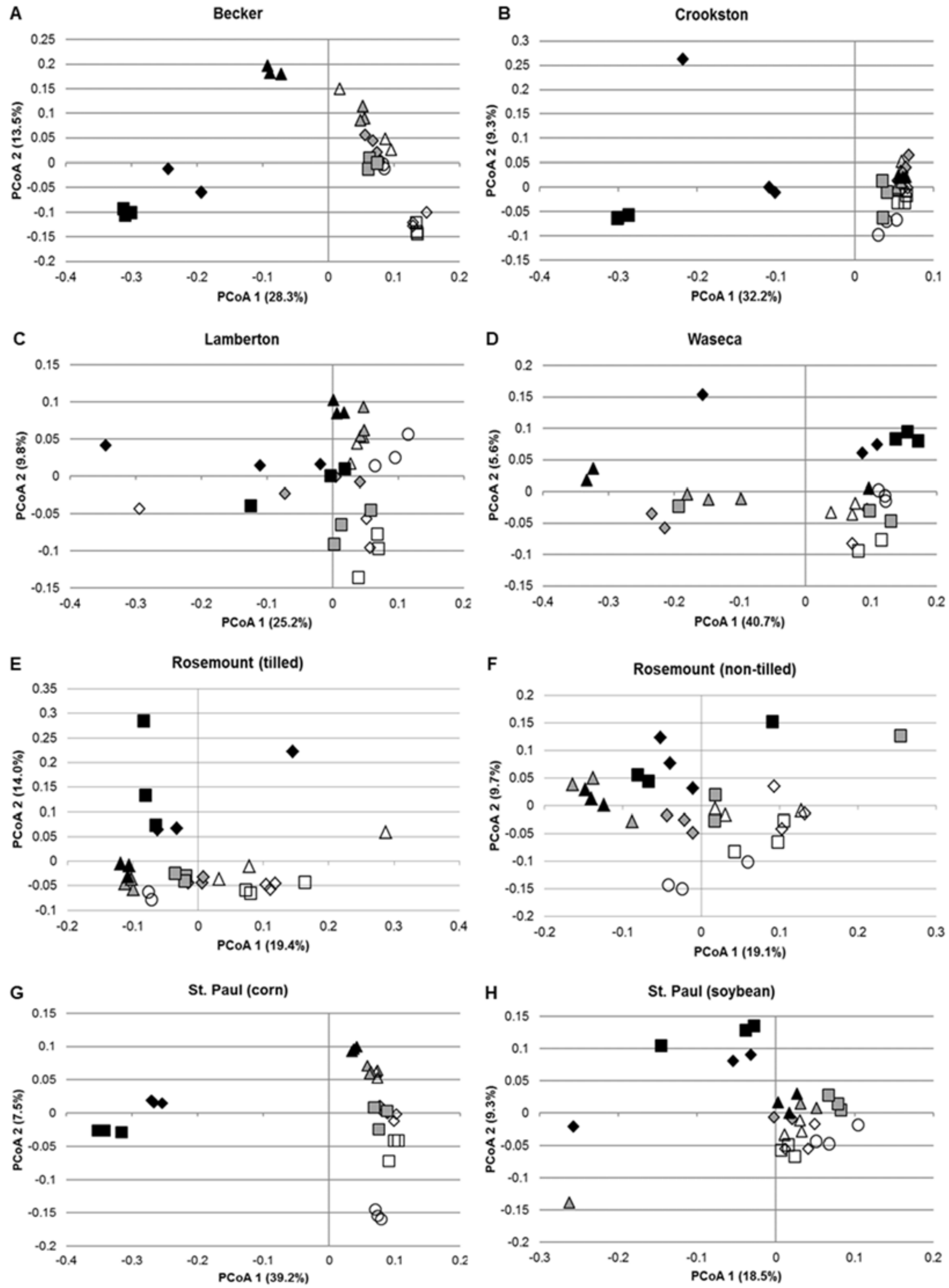

Figure S4 – PCoA of Bray-Curtis dissimilarity matrices among samples by site. A) Becker (r^2 = 0.93), B) Crookston (r^2 = 0.95), C) Lamberton (r^2 = 0.90), D) Waseca (r^2 = 0.94), E) Rosemount (tilled, r^2 = 0.89), F) Rosemount (non-tilled, r^2 = 0.79), G) St. Paul (corn, r^2 = 0.96), H) St. Paul (soybean, r^2 = 0.88). Shapes reflect time post-treatment: prior to treatment (○), 4 days (Δ), 14 days (◊), and 21 days (□) following treatment. Colors reflect treatment where open symbols were not amended, gray symbols were amended with 100 μg N g^{-1} soil, and black symbols were amended with 500 μg N g^{-1} soil.

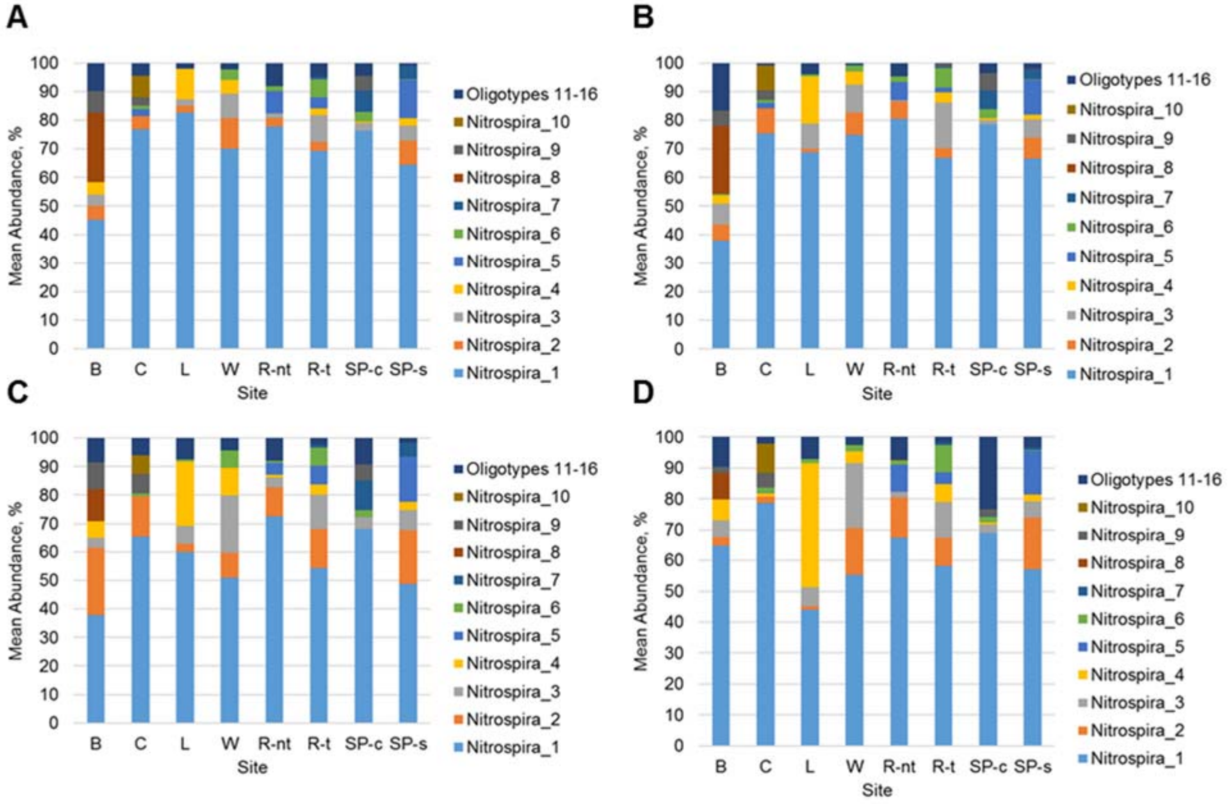

Figure S5 – Distribution of oligotypes within the genus *Nitrospira*. A) Initial sampling, prior to treatment, B) no urea amendment, day 21, C) 100 µg N g^{-1} soil amendment, day 21, D) 500 µg N g^{-1} soil amendment, day 21. Sampling sites include Becker (B); Crookston (C); Lamberton (L); Waseca (W); Rosemount (non-tilled, R-nt); Rosemount (tilled, R-t), St. Paul (corn, SP-c), and St. Paul (soybean, SP-s).

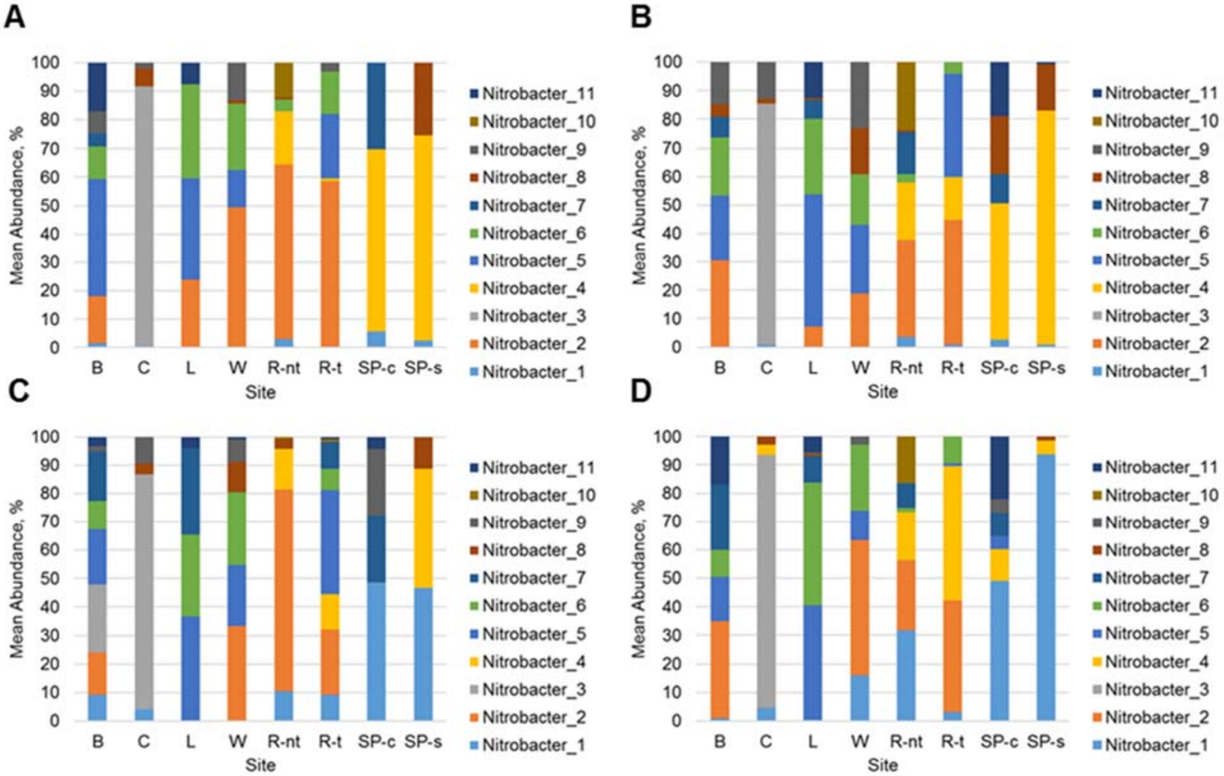

Figure S6 – Distribution of oligotypes within the genus *Nitrobacter*. A) Initial sampling, prior to treatment, B) no urea amendment, day 21, C) 100 μg N g^{-1} soil amendment, day 21, D) 500 μg N g^{-1} soil amendment, day 21. Sampling sites include Becker (B); Crookston (C); Lamberton (L); Waseca (W); Rosemount (non-tilled, R-nt); Rosemount (tilled, R-t), St. Paul (corn, SP-c), and St. Paul (soybean, SP-s).

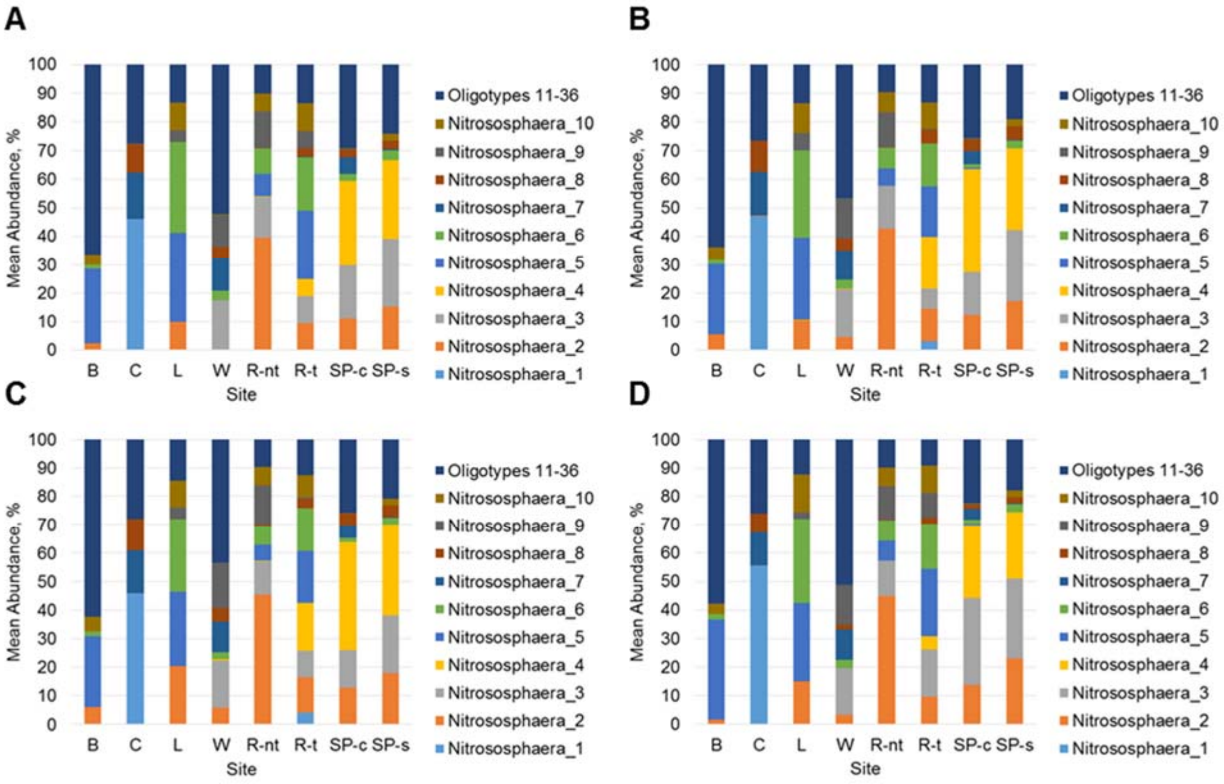

Figure S7 – Distribution of oligotypes within the genus *Nitrososphaera*. A) Initial sampling, prior to treatment, B) no urea amendment, day 21, C) 100 µg N g^{-1} soil amendment, day 21, D) 500 µg N g^{-1} soil amendment, day 21. Sampling sites include Becker (B); Crookston (C); Lamberton (L); Waseca (W); Rosemount (non-tilled, R-nt); Rosemount (tilled, R-t), St. Paul (corn, SP-c), and St. Paul (soybean, SP-s).

Table S1 – Edaphic parameters of soils prior to urea amendment.

Site	Soil composition (%)			pH	Organic matter	Organic N	Organic C	CEC*	K	Moisture
	Clay	Silt	Sand		(%)	(g N kg^{-1})	(g C kg^{-1})	(meq $100g^{-1}$)	(mg N L^{-1})	(g H_2O g^{-1})
Becker	11.5	7.7	80.8	7.4	4.5	0.93	12.6	8.1	207	0.161
Crookston	19.1	38.1	42.8	8.2	3.6	1.73	17.3	44.3	152	0.246
Lamberton	27.1	30.4	42.5	6.1	6.5	1.67	17.8	19.6	454	0.288
Waseca	29.6	33.2	37.2	6.6	8.4	2.39	30.2	36.7	169	0.321
Rosemount (tilled)	15.5	58.3	26.2	6.8	5.0	1.74	22.3	20.1	412	0.288
Rosemount (non-tilled)	10.4	55.6	34	6.7	7.0	2.29	28.6	21.2	254	0.298
Saint Paul (corn)	14.9	59.6	25.5	7.1	5.9	2.11	25.1	20.4	311	0.255
Saint Paul (soybean)	16.9	50.8	32.3	7.2	3.4	1.15	15.3	16.2	224	0.238

Data were previously reported in (Breuillin-Sessoms et al., 2017).

Table S2 – Log_{10}-transformed abundances of chemical analytes evaluated in this study. Values reflect mean ± standard deviation of triplicate samples collected at 21 d following urea amendment. All units, except where indicated, are μg N g^{-1} soil.

Site	Urea	log(CO_2)	log(H^+)	log(NH_4)*	log(NH_3)	log(NO_2)	log(NO_3)	log(N_2O)	log(HNO_2)
	0	-0.25 ± 0.60	-6.43 ± 0.06	-0.13 ± 0.07<br>*-0.64 ± 0.07*<br>-0.15 ± 0.07	-3.46 ± 0.06	-1.64 ± 0.01	1.43 ± 0.04	-0.25 ± 0.6	-3.97 ± 0.05
Becker	100	0.09 ± 0.27	-6.22 ± 0.07	-0.13 ± 0.03<br>*-0.64 ± 0.03*<br>-0.15 ± 0.03	-3.67 ± 0.09	-0.81 ± 0.15	2.07 ± 0.04	0.21 ± 0.05	-2.93 ± 0.08
	500	0.14 ± 0.11	-6.32 ± 0.05	2.40 ± 0.01<br>*2.07 ± 0.02*<br>2.37 ± 0.01	-0.85 ± 0.06	2.02 ± 0.05	1.91 ± 0.06	1.78 ± 0.1	-0.21 ± 0.04
	0	0.59 ± 0.41	-7.38 ± 0.06	-0.15 ± 0.05<br>*-1.10 ± 0.05*<br>-0.16 ± 0.05	-2.97 ± 0.06	-1.32 ± 0.15	1.19 ± 0.02	-0.12 ± 0.05	-4.79 ± 0.09
Crookston	100	0.49 ± 0.20	-7.52 ± 0.10	-0.14 ± 0.06<br>*-1.10 ± 0.06*<br>-0.16 ± 0.06	-2.82 ± 0.09	-1.27 ± 0.01	1.98 ± 0.00	0.23 ± 0.12	-4.88 ± 0.11
	500	0.94 ± 0.02	-7.63 ± 0.02	1.06 ± 0.10<br>*0.11 ± 0.10*<br>1.05 ± 0.10	-1.51 ± 0.09	0.64 ± 0.24	2.58 ± 0.02	0.42 ± 0.16	-3.08 ± 0.24
	0	0.52 ± 0.31	-5.39 ± 0.05	-0.01 ± 0.04<br>*-0.67 ± 0.04*<br>-0.03 ± 0.04	-4.52 ± 0.10	-1.33 ± 0.12	1.13 ± 0.05	-0.1 ± 0.06	-2.89 ± 0.08
Lamberton	100	0.55 ± 0.11	-4.92 ± 0.16	-0.01 ± 0.04<br>*-0.67 ± 0.04*<br>-0.04 ± 0.04	-4.99 ± 0.18	-1.30 ± 0.02	2.02 ± 0.02	0.18 ± 0.05	-2.39 ± 0.16
	500	0.50 ± 0.12	-4.49 ± 0.10	2.13 ± 0.01<br>*1.49 ± 0.01*<br>2.10 ± 0.01	-3.27 ± 0.10	-1.16 ± 0.24	2.51 ± 0.02	0.63 ± 0.06	-1.83 ± 0.32
Waseca	0‡	0.33 ± 0.54	-5.46 ± 0.33	0.13 ± 0.03<br>*-0.88 ± 0.03*<br>0.11 ± 0.03	-4.66 ± 0.35	-1.50 ± 0.01	1.39 ± 0.00	-0.44 ± 0.15	-3.17 ± 0.32

	100	0.16 ± 0.17	-5.66 ± 0.06	-0.16 ± 0.20<br>*-1.16 ± 0.20*<br>-0.17 ± 0.20	-4.75 ± 0.25	-0.69 ± 0.03	2.04 ± 0.03	0.43 ± 0.18	-2.55 ± 0.09
	500	0.61 ± 0.22	-5.11 ± 0.03	0.66 ± 0.03<br>*-0.34 ± 0.03*<br>0.65 ± 0.03	-4.47 ± 0.06	-0.01 ± 0.02	2.63 ± 0.03	0.48 ± 0.14	-1.33 ± 0.04
Rosemount (tilled)	0	-0.03 ± 0.21	-5.84 ± 0.09	0.03 ± 0.02<br>*-0.63 ± 0.02*<br>0.00 ± 0.02	-4.03 ± 0.11	-1.39 ± 0.09	1.35 ± 0.04	-0.16 ± 0.16	-3.38 ± 0.17
	100	0.91 ± 0.10	-6.42 ± 0.17	-0.02 ± 0.04<br>*-0.68 ± 0.04*<br>-0.05 ± 0.04	-3.50 ± 0.20	-0.59 ± 0.07	2.06 ± 0.04	0.05 ± 0.27	-3.17 ± 0.15
	500	0.82 ± 0.05	-6.12 ± 0.14	1.22 ± 0.26<br>*0.57 ± 0.26*<br>1.19 ± 0.26	-2.56 ± 0.22	-0.28 ± 0.32	2.58 ± 0.07	0.4 ± 0.33	-2.55 ± 0.42
Rosemount (non-tilled)	0	-0.37 ± 0.51	-5.94 ± 0.14	0.05 ± 0.07<br>*-0.58 ± 0.07*<br>0.02 ± 0.07	-3.89 ± 0.07	-1.46 ± 0.04	1.63 ± 0.02	-0.25 ± 0.23	-3.57 ± 0.11
	100	1.23 ± 0.15	-6.84 ± 0.21	-0.01 ± 0.02<br>*-0.65 ± 0.02*<br>-0.05 ± 0.02	-3.06 ± 0.20	-0.52 ± 0.10	2.07 ± 0.02	0.39 ± 0.1	-3.53 ± 0.30
	500	0.93 ± 0.03	-6.41 ± 0.20	1.47 ± 0.08<br>*0.85 ± 0.08*<br>1.44 ± 0.08	-1.99 ± 0.27	-0.47 ± 0.08	2.63 ± 0.00	0.42 ± 0.03	-3.05 ± 0.13
St. Paul (corn)	0	-0.02 ± 0.11	-6.42 ± 0.04	-0.06 ± 0.02<br>*-0.70 ± 0.02*<br>-0.09 ± 0.02	-3.52 ± 0.05	-0.85 ± 0.16	1.19 ± 0.05	-0.19 ± 0.12	-3.38 ± 0.19
	100	-0.59 ± 0.30	-6.2 ± 0.02	-0.13 ± 0.05<br>*-0.77 ± 0.05*<br>-0.16 ± 0.05	-3.81 ± 0.07	-0.55 ± 0.19	1.87 ± 0.01	-0.16 ± 0.42	-2.86 ± 0.19
	500	-0.2 ± 0.11	-5.7 ± 0.07	1.14 ± 0.01<br>*0.50 ± 0.01*<br>1.11 ± 0.01	-3.04 ± 0.06	-0.15 ± 0.06	2.43 ± 0.00	0.42 ± 0.04	-1.96 ± 0.12

St. Paul (soybean)	0	0.02 ± 0.27	-6.04 ± 0.04	0.15 ± 0.04<br>*-0.51 ± 0.04*<br>0.12 ± 0.04	-3.71 ± 0.04	-1.48 ± 0.01	1.23 ± 0.01	0.05 ± 0.08	-3.6 ± 0.03
	100	0.43 ± 0.12	-5.86 ± 0.03	-0.13 ± 0.12<br>*-0.78 ± 0.12*<br>-0.15 ± 0.12	-4.17 ± 0.15	-0.60 ± 0.03	1.99 ± 0.00	0.28 ± 0.10	-2.54 ± 0.00
	500	0.73 ± 0.11	-5.57 ± 0.03	2.06 ± 0.02<br>*1.46 ± 0.03*<br>2.04 ± 0.02	-2.21 ± 0.05	-0.39 ± 0.56	2.51 ± 0.01	0.03 ± 0.36	-2.04 ± 0.57

Data were previously reported in (Breuillin-Sessoms et al., 2017).

[*]Values refer to total extractable NH_4^+ (μg N g^{-1}), solution-phase NH_4^+ is shown in italics (*sl*NH_4^+; mg N L^{-1}), and sorbed NH_4^+ (*sr*NH_4^+; mg N kg^{-1}) is shown underlined.

[†]Values reflect only two replicates.

Table S3 – Log_{10}-transformed abundances of genes evaluated and previously reported in (Breuillin-Sessoms et al., 2017). Values are mean ± standard deviation of triplicate samples and reflect gene abundances measured from samples collected at 21 d following urea amendment.

Site	Urea amendment (μg N g^{-1} soil)	log(16S)	log(*amoA*)*	log(*nxrA*)	log(*nxrB*)
Becker	0	6.68 ± 0.05	3.82 ± 0.11	3.96 ± 0.07	5.06 ± 0.08
	100	6.58 ± 0.07	4.67 ± 0.05	4.56 ± 0.05	5.02 ± 0.04
	500	6.56 ± 0.05	5.37 ± 0.10	3.91 ± 0.08	4.48 ± 0.13
Crookston	0	6.17 ± 0.06	3.93 ± 0.02	4.01 ± 0.04	4.99 ± 0.05
	100	7.00 ± 0.02	4.02 ± 0.10	4.31 ± 0.11	4.79 ± 0.13
	500	7.08 ± 0.06	5.02 ± 0.04	4.13 ± 0.07	4.55 ± 0.04
Lamberton	0	6.22 ± 0.12	3.70 ± 0.04	4.26 ± 0.01	4.62 ± 0.06
	100	6.39 ± 0.04	4.39 ± 0.04	4.35 ± 0.03	4.51 ± 0.02
	500	6.39 ± 0.16	5.28 ± 0.20	4.75 ± 0.14	4.60 ± 0.11
Waseca	0†	6.56 ± 0.02	3.82 ± 0.02	3.82 ± 0.01	4.42 ± 0.02
	100	7.04 ± 0.05	4.40 ± 0.12	4.69 ± 0.00	4.52 ± 0.04
	500	6.99 ± 0.06	4.74 ± 0.07	5.00 ± 0.02	4.35 ± 0.07
Rosemount (tilled)	0	6.09 ± 0.09	3.25 ± 0.14	3.52 ± 0.10	4.54 ± 0.20
	100	6.69 ± 0.20	4.79 ± 0.16	4.81 ± 0.28	4.81 ± 0.22
	500	6.35 ± 0.20	5.58 ± 0.43	4.34 ± 0.61	4.35 ± 0.21
Rosemount (non-tilled)	0	6.36 ± 0.02	3.68 ± 0.02	3.29 ± 0.01	4.38 ± 0.03
	100	6.44 ± 0.22	4.60 ± 0.25	4.36 ± 0.21	5.03 ± 0.24
	500	6.70 ± 0.33	5.33 ± 0.22	4.83 ± 0.20	5.12 ± 0.28
St. Paul (corn)	0	6.82 ± 0.01	3.67 ± 0.13	3.86 ± 0.05	5.02 ± 0.06
	100	6.79 ± 0.05	4.08 ± 0.07	3.94 ± 0.05	5.27 ± 0.07
	500	6.84 ± 0.10	4.75 ± 0.17	3.88 ± 0.06	5.07 ± 0.03
St. Paul (soybean)	0	6.55 ± 0.06	3.69 ± 0.03	3.98 ± 0.60	4.92 ± 0.05
	100	6.78 ± 0.05	4.38 ± 0.06	4.84 ± 0.03	4.63 ± 0.11
	500	6.71 ± 0.07	5.46 ± 0.05	5.28 ± 0.06	4.80 ± 0.07

*Only *amoA* from bacteria are represented.
†Values reflect only two replicates.

References

Breuillin-Sessoms, F., Venterea, R. T., Sadowsky, M. J., Coulter, J. A., Clough, T. J., and Wang, P. (2017). Nitrification gene ratio and free ammonia explain nitrite and nitrous oxide production in urea-amended soils. *Soil Biol. Biochem.* 111, 143–153. doi:10.1016/j.soilbio.2017.04.007.

Mulvaney, R. L. (1996). "Nitrogen-inorganic forms", in *Methods of Soil Analysis*, ed. D. L. Sparks (Madison, WI: American Society of Agronomy), 1123–1184.

Stevens, R. J., and Laughlin, R. J. (1995). Nitrite transformations during soil extraction with potassium chloride. *Soil Sci. Soc. Am. J.* 59, 933. doi:10.2136/sssaj1995.03615995005900030044x.

Venterea, R. T., Clough, T. J., Coulter, J. A., Breuillin-Sessoms, F., Wang, P., and Sadowsky, M. J. (2015). Ammonium sorption and ammonia inhibition of nitrite-oxidizing bacteria explain contrasting soil N_2O production. *Sci Rep* 5, 12153. doi:10.1038/srep12153.

Venterea, R. T., and Rolston, D. E. (2000). Nitric and nitrous oxide emissions following fertilizer application to agricultural soil: Biotic and abiotic mechanisms and kinetics. *J. Geophys. Res.* 105, 15117. doi:10.1029/2000JD900025.
